# Hsp70-nucleotide exchange factor (NEF) Fes1 has non-NEF roles in degradation of gluconeogenic enzymes and cell wall integrity

**DOI:** 10.1371/journal.pgen.1008219

**Published:** 2019-06-26

**Authors:** Shailesh Kumar, Daniel C. Masison

**Affiliations:** Laboratory of Biochemistry and Genetics, National Institute of Diabetes and Digestive and Kidney Diseases, National Institutes of Health, Bethesda, Maryland, United States of America; Centre National de la Recherche Scientifique, FRANCE

## Abstract

Fes1 is a conserved armadillo repeat-containing Hsp70 nucleotide exchange factor important for growth at high temperature, proteasomal protein degradation and prion propagation. Depleting or mutating Fes1 induces a stress response and causes defects in these processes that are ascribed solely to disruption of Fes1 regulation of Hsp70. Here, we find Fes1 was essential for degradation of gluconeogenic enzymes by the vacuole import and degradation (Vid) pathway and for cell wall integrity (CWI), which is crucial for growth at high temperature. Unexpectedly, Fes1 mutants defective in physical or functional interaction with Hsp70 retained activities that support Vid and CWI. Fes1 and the Fes1 mutants bound to the Vid substrate Fbp1 in vitro and captured Slt2, a signaling kinase that regulates CWI, from cell lysates. Our data show that the armadillo domain of Fes1 binds proteins other than Hsp70, that Fes1 has important Hsp70-independent roles in the cell, and that major growth defects caused by depleting Fes1 are due to loss of these functions rather than to loss of Hsp70 regulation. We uncovered diverse functions of Fes1 beyond its defined role in regulating Hsp70, which points to possible multi-functionality among its conserved counterparts in other organisms or organelles.

## Author summary

Fes1, a yeast homolog of human nucleotide exchange factor HspBP1, binds and regulates Hsp70, a universally conserved protein that helps maintain health of proteins in cells. Fes1 is believed to function only by helping Hsp70 release ADP and substrates and cells lacking Fes1 are sick. We find Fes1 is essential for protein degradation by a vacuolar pathway (Vid) and for cell wall integrity (CWI), and it interacts with a Vid substrate and a regulator of CWI. Fes1 mutants that cannot regulate Hsp70 can still support Vid and CWI, interact with proteins involved in these processes and restore cell health. Thus, Fes1 binds proteins other than Hsp70 and has important functions beyond regulating Hsp70 that are needed for optimal cell fitness.

## Introduction

Fbp1 and other gluconeogenic enzymes that are highly expressed when cells are starved of glucose are rapidly inactivated and degraded when glucose is restored. Restoring glucose after starving one day causes Fbp1 to be degraded by the proteasome [1,2], but when cells are starved three days before restoring glucose it is imported into specialized vesicles that transit to the vacuole in a process called vacuole import and degradation (Vid) [3–8]. Import of Fbp1 into Vid vesicles requires the cytosolic Hsp70 Ssa2 [9]. The nearly identical Ssa1 cannot substitute for Ssa2 in this process, but swapping one non-conserved amino acid (Ala83/Gly83) between them is enough to switch their ability to function in the Vid pathway [10].

Hsp70s act in protein folding and transport by binding and releasing exposed hydrophobic surfaces of proteins in a two-step ATP-regulated cycle. ATP-bound Hsp70 is in an "open" substrate-accessible state. ATP hydrolysis causes a lid-like structure to close over bound substrates, effectively trapping them. Release of ADP and ensuing rebinding of ATP facilitates return to the open state and release of substrates. The low intrinsic rate of this cycle primes Hsp70 for rigorous regulation by many co-chaperones. In particular, J-proteins and nucleotide exchange factors (NEFs) are key Hsp70 partners that regulate the nucleotide hydrolysis and exchange steps, respectively [11]. J-proteins recruit Hsp70 to various locations, present substrates to Hsp70 and promote ATP hydrolysis. NEFs accelerate dissociation of ADP to facilitate release of substrates. Yeast have twenty-two J-proteins and four cytosolic NEFs [12,13] that cooperate to regulate this Hsp70 cycle, and the many possible combinations of these and other regulators provide both specificity and extensive versatility to Hsp70 function.

Ssa1/Ssa2 residue 83 is near a region where NEFs interact [14], so we hypothesized the specificity of this residue for Vid function was mediated by a difference in physical or functional interaction with NEFs. Here we tested if Hsp70 NEFs dictated specificity of Ssa2 for Vid function and found the NEF Fes1 [15] was itself required for Vid degradation of Fbp1. Yet, differences in physical interaction of Fes1 with Ssa1 and Ssa2 did not seem significant enough to account for the specificity in Vid function. Unexpectedly, when we tested whether Fes1 affected ability of Ssa1 or Ssa2 to bind Fbp1, we found Fes1 itself bound Fbp1, showing it can bind a protein other than Hsp70.

Fes1, which is related to human NEF HspBP1, plays a role in helping Hsp70 deliver substrates to the proteasome and cells lacking Fes1 display reduced proteasome activity, a constitutively activated stress response and temperature sensitivity [15,16]. Fes1 has an armadillo repeat domain important for binding Hsp70 to facilitate release of ADP and a "release domain" that occupies the Hsp70 substrate-binding pocket to ensure released substrates do not rebind [14,16,17]. Because mutants of Fes1 defective in either of these functions failed to provide Fes1 function in vivo, the role of Fes1 is thought to be limited to its regulation of Hsp70. Our initial findings prompted us to re-evaluate cellular functions of such Fes1 mutants. We found they not only retained Fes1 functions important for Vid and for growth at elevated temperature, but also bound to Fbp1 and provided functions important for cell wall integrity (CWI) and growth under various stresses. Our findings uncover unanticipated Fes1 substrate-binding activity and imply that Fes1 can perform important Hsp70-independent functions in cells.

## Results

### Fes1 is required for Fbp1 degradation by Vid

**Fig 1:**
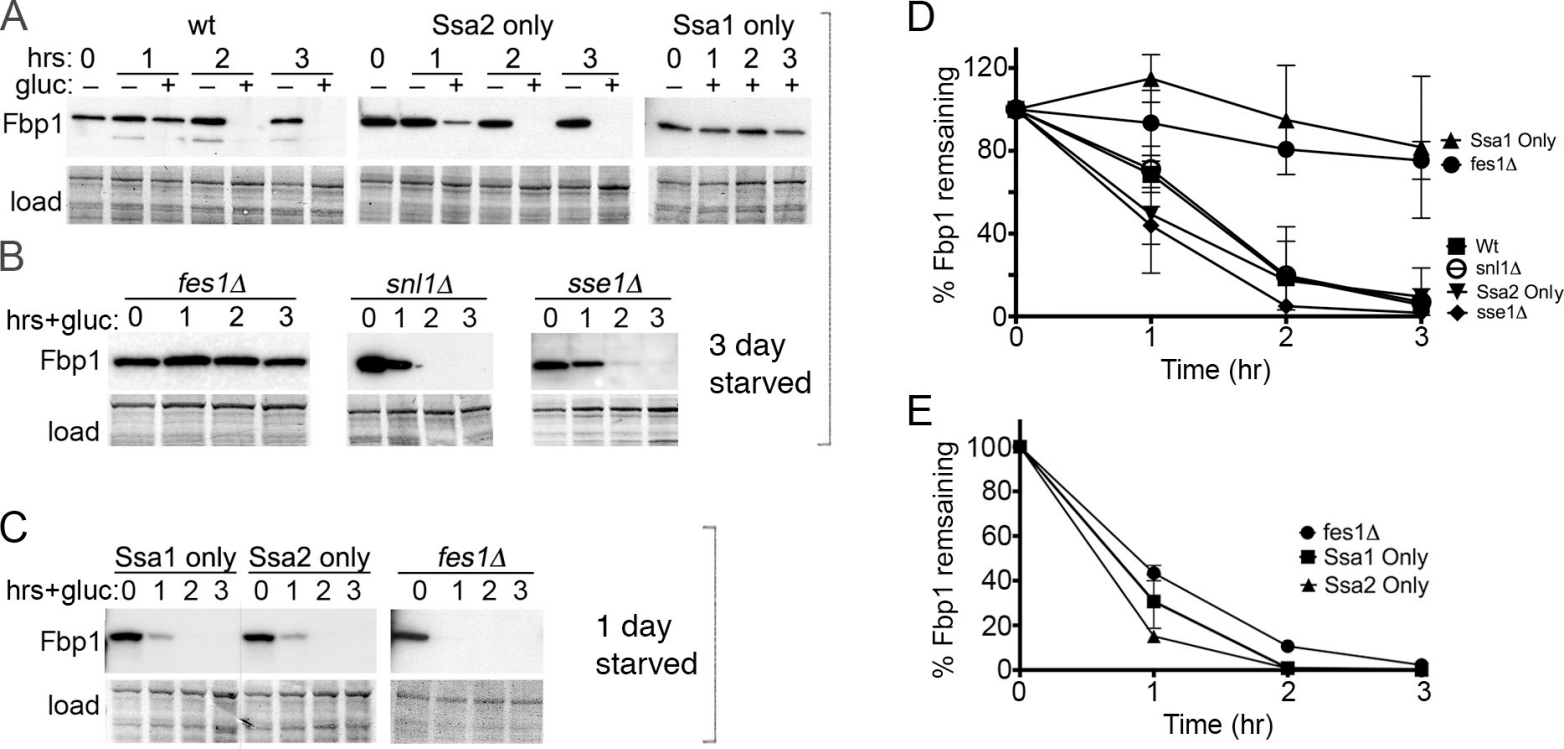
Fes1 is required for degradation of Fbp1 by Vid. Panels A-C show immunoblots probing for Fbp1. (A) Cells starved of glucose for three days were transferred to glucose-rich medium, which triggers Vid, and Fbp1 abundance was monitored by western analysis for three hours. Strains are 1075 (wt), SY136 (center) or SY135 (right). SY136 and SY135 express Ssa2 or Ssa1, respectively, as the only Ssa Hsp70 in the cell. Image labeled "load" shows the blotted membrane stained by amido-black as loading and transfer controls. (B) As in panel (A) using strains SY346, SY351 and BY241ΔSSE, which lack NEFs Fes1, Snl1 or Sse1 as indicated. (C) As in panel (A) except glucose was restored after starving cells for one day, which triggers degradation of Fbp1 by proteasomes. (D, E) Plots of data represented by experiments in panels (A-C) quantified using Image-J from at least 3 independent replicates of strains starved for three days (D) or one day (E). Error bars represent SD.

Restoring glucose to cells starved of glucose for three days causes rapid degradation of Fbp1 by Vid [18]. After Vid activation, the abundance of Fbp1 in our wild type cells was noticeably reduced after an hour, and by two hours it was barely detectable (Fig 1A). As seen before [9,10], this degradation depended on a function of Ssa2 that cannot be provided by Ssa1.

Our earlier findings suggested that this difference between Ssa1 and Ssa2 in the Vid pathway could be due to differential interaction of the Hsp70s with NEFs [10]. The yeast cytosolic Hsp70 NEFs are Fes1, Snl1, Sse1 and its paralog Sse2. We used strains lacking individual NEFs to test if any of them have a role in Vid. Because deleting *SSE1* causes pleiotropic effects, deleting *SSE2* has no overt phenotype and deleting both is lethal [19–21], we used cells lacking only *SSE1* to test depletion of Sse NEF function. We found degradation of Fbp1 by Vid was normal in cells lacking Snl1 or Sse1, but was severely impaired in *fes1Δ* cells (Fig 1B and 1D). Thus, Fes1, but not the other NEFs, has an essential role in degradation of Fbp1 by the Vid pathway.

We then monitored proteasomal degradation of Fbp1 by doing similar experiments using cells that were starved for only one day before restoring glucose (Fig 1C and 1E). This proteasomal degradation of Fbp1 was rapid and did not depend on Ssa2 or on Fes1, as seen by others [9,16].

### Fes1 interacts similarly with Ssa1 and Ssa2 and binds Fbp1

**Fig 2:**
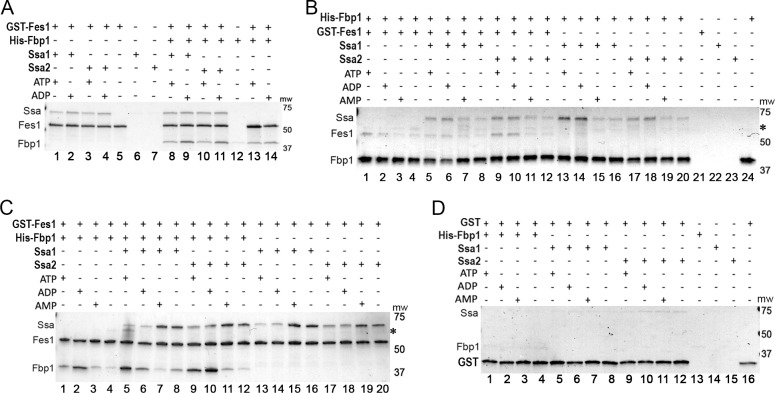
Fes1 interacts similarly with Ssa1 and Ssa2 and binds Fbp1. All panels show Coomassie-stained SDS-PAGE gels of pull-down reactions where protein pulled down is indicated on top row. (A) GST-Fes1 was pulled down using glutathione resin. Fes1 binds Ssa1 and Ssa2 similarly (lanes 1–4), binds Fbp1 separately (lanes 13–14) and captures both Hsp70 and Fbp1 when all three proteins are present (lanes 8–11). (B) As in panel (A) except His6-Fbp1 was pulled down by metal affinity and reactions with AMP or no nucleotides were included. Fbp1 captures Fes1 (lanes 1–4) and Hsp70 (lanes 13–20) separately and captures both when all three proteins are mixed (lanes 5–12). (C) As in panel (A) expanded to include reactions with AMP or without nucleotide. (D) Control reactions using GST alone in place of GST-Fes1. Asterisks in panels B and C indicate position of contaminant sometimes found in Fbp1 preparations.

Fes1 is known only as a regulator of Hsp70, so these results suggested that Fes1 underlies the requirement of Ssa2 for Vid. We first tested this idea by looking for differences in interaction of Fes1 with Ssa1 and Ssa2 using purified proteins. After mixing GST-Fes1 with the individual Hsp70s we pulled down Fes1 on glutathione resin and assessed its ability to capture Hsp70 (Fig 2A). As ATP and ADP bind Hsp70 and regulate its substrate-binding cycle, we included these nucleotides separately in the reactions. To test if Fes1 affected interactions of Ssa1 or Ssa2 with Fbp1, we performed a similar set of reactions that also contained His6-Fbp1.

Fes1 captured similar amounts of Ssa1 and Ssa2 in reactions without Fbp1 (Fig 2A, lanes 1–4) or with Fbp1 (Fig 2A, lanes 8–11). Fes1 bound slightly more Hsp70 in reactions with ADP than in those with ATP, as seen by others [15]. This difference was observed consistently in our pull-down reactions. In the reactions that included Fbp1, both Fbp1 and the Hsp70s were captured, which we presumed could occur in a ternary complex with Fes1 binding Hsp70 as NEF and Fbp1 being bound to Hsp70 as substrate. Unexpectedly, however, Fes1 bound Fbp1 in control reactions without Hsp70 (Fig 2A, lanes 13 and 14). Only traces of the Hsp70s (Fig 2A, lanes 6–7) or Fbp1 (lane 12) bound to the column when Fes1 was omitted. Together these results suggest that the specificity of Ssa2 for Vid is not determined by a difference in the way Fes1 physically interacts with Ssa1 and Ssa2, and show that Fes1 can bind Fbp1 directly.

To substantiate these conclusions, we performed similar experiments with the same proteins, but instead pulled down His6-Fbp1 on metal affinity resin and assessed its ability to capture Hsp70 and Fes1 (Fig 2B). Although ATP and ADP can bind Fbp1 and inhibit its activity, AMP is recognized as a primary allosteric inhibitor of Fbp1 [22,23], raising the possibility that it might influence binding of Fes1 to Fbp1. We therefore included additional reactions containing AMP. In agreement with our results above, His6-Fbp1 captured Fes1 when Hsp70 was absent (Fig 2B, lanes 1–4), which confirms Hsp70-independent binding of Fes1 to Fbp1. Compared with reactions lacking nucleotide, addition of AMP did not affect this binding, but ATP and ADP each enhanced it. This nucleotide dependency of Fes1-Fbp1 interaction was consistent in all our pull-down experiments, regardless of which protein was pulled down. His6-Fbp1 also bound Hsp70 (Ssa1 and Ssa2) when Fes1 was omitted (Fig 2B, lanes 13–20), and this binding also occurred best when ATP or ADP was present. When all three proteins were mixed (Fig 2B, lanes 5–12) His6-Fbp1 bound both Hsp70 and Fes1 in similar proportions and with similar nucleotide dependencies as when they were in separate reactions. These complementary experiments show that Fes1 and Fbp1 interact directly with Hsp70 and with each other.

We then repeated the experiment using GST-Fes1, as in Fig 2A, but added reactions with AMP or without nucleotides (Fig 2C). In agreement with the way Fbp1 pulled down Fes1 (Fig 2B), capture of Fbp1 by GST-Fes1 was more effective when ATP or ADP was present (Fig 2C, lanes 1–4), confirming that ATP and ADP enhanced binding of Fes1 to Fbp1. Also in line with our initial observations in Fig 2A, GST-Fes1 bound Hsp70 (both Ssa1 and Ssa2) in the presence of ATP and ADP (Fig 2C, lanes 13–20). Unexpectedly, Fes1 captured more Hsp70 in reactions with AMP or without nucleotides. Use of these binding conditions has not been reported previously, presumably because AMP has no known role in Hsp70 function and it is unclear why Fes1 would bind a nucleotide-free state of Hsp70. The physiological relevance of these results is unclear, but we observed this pattern of nucleotide influence on Hsp70 capture by GST-Fes1 consistently in reactions with or without Fbp1. In reactions containing all three proteins, GST-Fes1 captured Hsp70 best with AMP or without nucleotides and captured Fbp1 best with ATP and ADP (Fig 2C, lanes 5–12), which is consistent with the way it bound Hsp70 and Fbp1 in separate reactions.

As additional controls for non-specific binding we repeated the pull down using GST alone in place of GST-Fes1 and found that only traces of Hsp70 and Fbp1 bound to the column and that the presence or absence of nucleotides did not affect the amounts of proteins bound (Fig 2D). Therefore, the Fbp1 and Hsp70 that was captured by GST-Fes1 was binding primarily to Fes1 and not to GST.

### Fbp1 residues 2–12 are not involved in binding to Fes1

The first 12 amino acids of Fbp1 and other gluconeogenic enzymes, particularly a conserved proline at position 2, are needed for their degradation by both the Vid and proteasome pathways [7,24,25]. Additionally, serine at position 12 of Fbp1 is phosphorylated, although this modification is not essential for Fbp1 degradation by Vid, and threonine residue 13 is a potential phosphorylation site [7,26,27]. Fes1 is dispensable for proteasomal degradation of Fbp1 (Fig 1 and [16]), but this region of Fbp1 might contribute to the Vid requirement of Fes1 or Ssa2. We tested if these Fbp1 residues were important for it to bind Fes1 or Hsp70 by deleting or mutating them.

Deleting P2, mutating it to alanine (P2A), or combining P2A with S12A and T13A all had little effect on ability of purified His6-Fbp1 to capture Ssa1, Ssa2 or Fes1 in vitro (S1A and S1B Fig). His6-Fbp1Δ2–12, which lacks amino acid residues 2–12, also captured both Fes1 and Hsp70 (S1C Fig). In a complementary experiment, GST-Fes1 captured both Fbp1Δ2–12 and Hsp70 (S1D Fig). These results suggest that the role of residues 2–12 for the degradation of Fbp1 by Vid is not to mediate an interaction with Hsp70 or Fes1.

### Role of Fes1 in the Vid pathway is independent of its NEF activity

**Fig 3:**
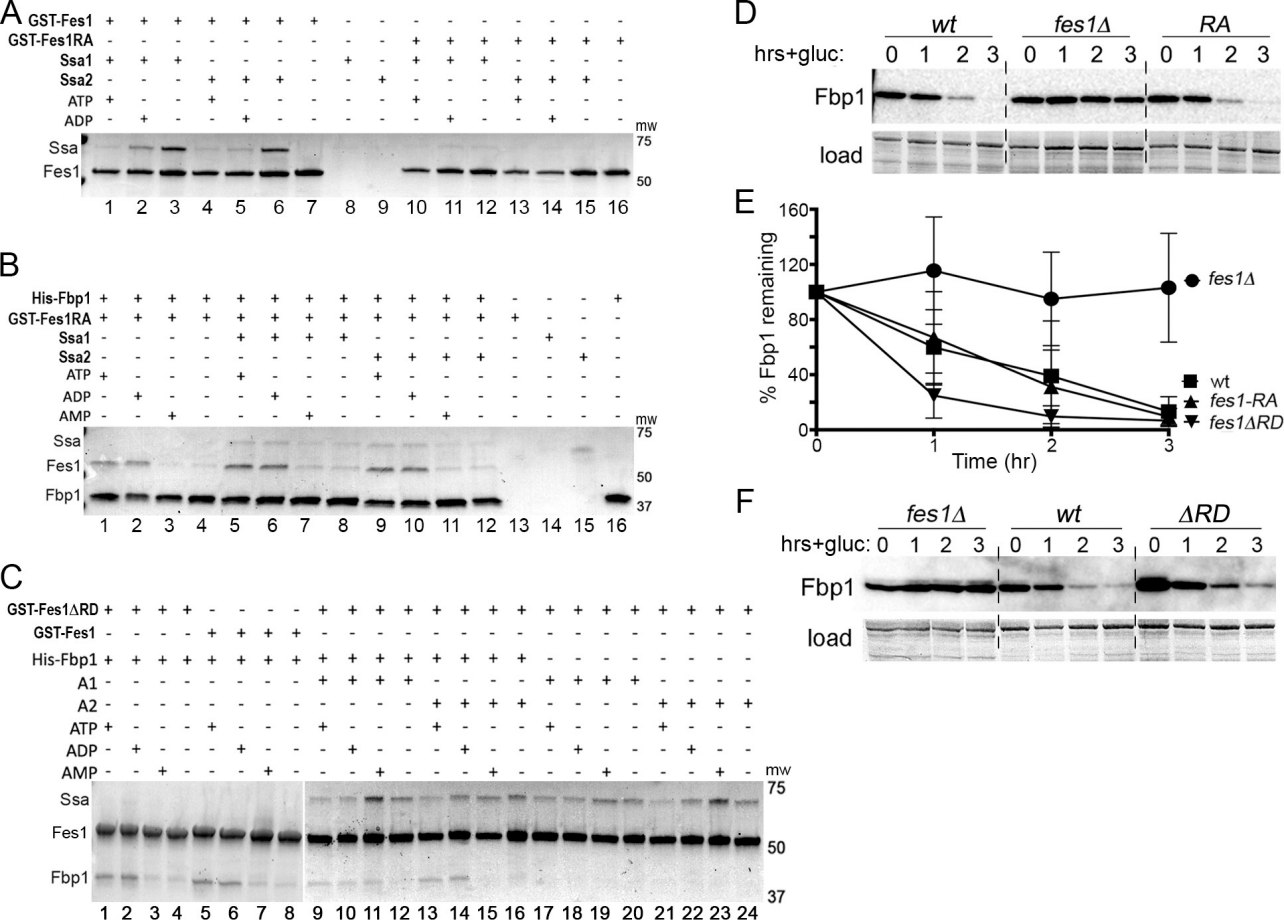
Fes1^A79R,R195A^ binds Fbp1, but not Hsp70; FesΔRD binds Fbp1 and Hsp70; both Fes1^A79R,R195A^ and FesΔRD function in Vid. Panels (A-C) show Coomassie-stained SDS-PAGE gels where protein pulled down is indicated on top rows; panels (D) and (F) are immunoblots probing for Fbp1. (A) GST-Fes1 (lanes 1–7) or GST-Fes1^A79R,R195A^ (Fes1RA, lanes 10–16) was pulled down using glutathione resin. (B) As in panel (A), but His6-Fbp1 was pulled down using metal affinity. The spot on lane 15 is a smudge; it is diffuse, does not fill lane width and is in a position that does not align with any protein. (C) As in Fig 2C, but GST-Fes1ΔRD was pulled down, except for lanes 5–8 where GST-Fes1 was used for direct comparison to lanes 1–4. Gap separating lanes 1–8 from 9–24 indicate separate gels. (D) Vid assay as in Fig 1, panel A, showing Fbp1 is degraded efficiently by Vid in cells expressing Fes1^A79R,R195A^ (strain SKY207, *RA*). (E) Plots of data from experiments represented in panels D and F quantified using Image-J from at least three replicates of each strain. Error bars represent SD. (F) As in (D), showing Fbp1 degradation by Vid in cells expressing Fes1ΔRD (strain 1853–35, *ΔRD*).

Fes1 residues A79 and R195 are needed for physical interaction of Fes1 with Hsp70 [14] and it was shown that the Fes1^A79R,R195A^ double mutant does not interact with Hsp70 in vitro or provide Fes1 function in vivo [14,16,28,29]. In repeating the in vitro experiments, we found both Ssa1 and Ssa2 were clearly captured by wild type GST-Fes1 (Fig 3A, lanes 1–6), but these Hsp70s were observed in only trace amounts from reactions pulling down GST-Fes1^A79R,R195A^ (lanes 10–15). Differences in quantified ratios of GST-Fes1^A79R,R195A^/Hsp70 in this gel and those of the background control GST/Hsp70 in Fig 2D lanes 5–12 were negligible, indicating that any Hsp70 detected in these reactions was likely not from binding to Fes1^A79R,R195A^. These results agree with the earlier conclusion that Fes1^A79R,R195A^ does not bind Ssa1 in vitro and here we show it similarly fails to bind Ssa2.

In contrast, His6-Fbp1 captured GST-Fes1^A79R,R195A^ in reactions without Hsp70 (Fig 3B, lanes 1–4), or with Hsp70 (lanes 5–12) showing again that the combined A79R and R195A mutations do not prevent binding of Fes1 to Fbp1. In all reactions containing His6-Fbp1 and GST-Fes1^A79R,R195A^, binding of His6-Fbp1 to GST-Fes1^A79R,R195A^ showed similar nucleotide dependence as with wild type GST-Fes1 (compare Fig 3B with Fig 2B). As in reactions with wild type GST-Fes1, His6-Fbp1 again captured more Hsp70 in reactions with ATP and ADP compared with those containing AMP or without nucleotides. Thus, Fbp1 bound to Fes1^A79R,R195A^ in a pattern similar to that of wild type Fes1.

When comparing relative amounts of Fes1^A79R,R195A^ and wild type Fes1 that were pulled down by His6-Fbp1 in reactions with Hsp70, however, it was apparent that Fbp1 captured more Fes1^A79R,R195A^ than wild type Fes1 (compare differences in amounts of Fes1 and Hsp70 in Fig 3B, lanes 5–12 with those in Fig 2B, lanes 5–12). This visually evident difference was confirmed by quantifying relative amounts of Fes1 and Hsp70 in these gels (Table 1). This difference could be explained simply by there being more Fes1^A79R,R195A^ available to bind His6-Fbp1 because it is not bound to Hsp70.

**Table 1:** Ratios of amounts of Fes1 and Hsp70 pulled down by Fbp1.

	Ratio of amounts of proteins in reactions containing:			
Proteins	ATP	ADP	AMP	none
Fes1/Ssa1	0.58 (± 0.11)	0.50 (± 0.08)	0.18 (± 0.01)	0.23 (± 0.05)
Fes1^A79R,R195A^/Ssa1	6.4 (± 1.6)	5.0 (± 1.5)	3.3 (± 3.0)	1.7 (± 0.50)
Fes1/Ssa2	0.79 (± 0.17)	0.67 (± 0.04)	0.32 (± 0.04)	0.33 (± 0.09)
Fes1^A79R,R195A^/Ssa2	4.3 (± 1.1)	4.5 (± 0.10)	1.9 (± 1.2)	2.4 (± 0.35)

Density of protein bands from images in Fig 2B (Fes1) and Fig 5B (Fes1^A79R,R195A^) were quantified using Image J software. Values are averages of two independent experiments (± range).

The first 34 amino acids of Fes1 is defined as a release domain (RD) important for ensuring substrate release by Hsp70 [17]. After Fes1 promotes nucleotide exchange and substrate dissociates from Hsp70, the RD occupies the Hsp70 substrate-binding pocket to prevent rebinding of substrates. Fes1ΔRD, which lacks residues 2–34, still binds Hsp70 and has NEF activity in vitro, but it lacks this substrate mimic function and Fes1ΔRD does not complement growth or proteasome defects of *fes1Δ* cells [17]. We repeated the in vitro pull-down reactions using GST-Fes1ΔRD and found it captured Hsp70 and Fbp1 in relative amounts and with nucleotide dependencies that were similar to those of wild type GST-Fes1 (Fig 3C, compare lanes 1–4 with lanes 5–8, and lanes 9–24 with Fig 2C, lanes 5–20). These results indicate that the armadillo domain of Fes1 is enough to confer the wild type pattern of interactions with both proteins.

If the essential role of Fes1 in Vid were mediated by its ability to regulate Hsp70, then degradation of Fbp1 should be impaired in cells expressing Fes1^A79R,R195A^ in place of wild type Fes1. We found, however, that Vid degradation of Fbp1 in strain SKY207, which expresses Fes1^A79R,R195A^ from the *FES1* genomic locus, was as efficient as that in wild type cells (Fig 3D and 3E). These results suggest that the requirement of Fes1 for Vid does not depend on interaction of Fes1 with Hsp70 and that Fes1^A79R,R195A^ retains Fes1 function needed for Vid that is separate from its NEF regulation of Hsp70. We also constructed a strain (1853–35) expressing Fes1ΔRD from the native *FES1* locus and found Fbp1 was degraded effectively by Vid in this strain (Fig 3E and 3F). Thus, the RD function of Fes1 was also dispensable for Vid.

### Fes1 NEF mutant retains Fes1 functions in cells grown under stress

**Fig 4:**
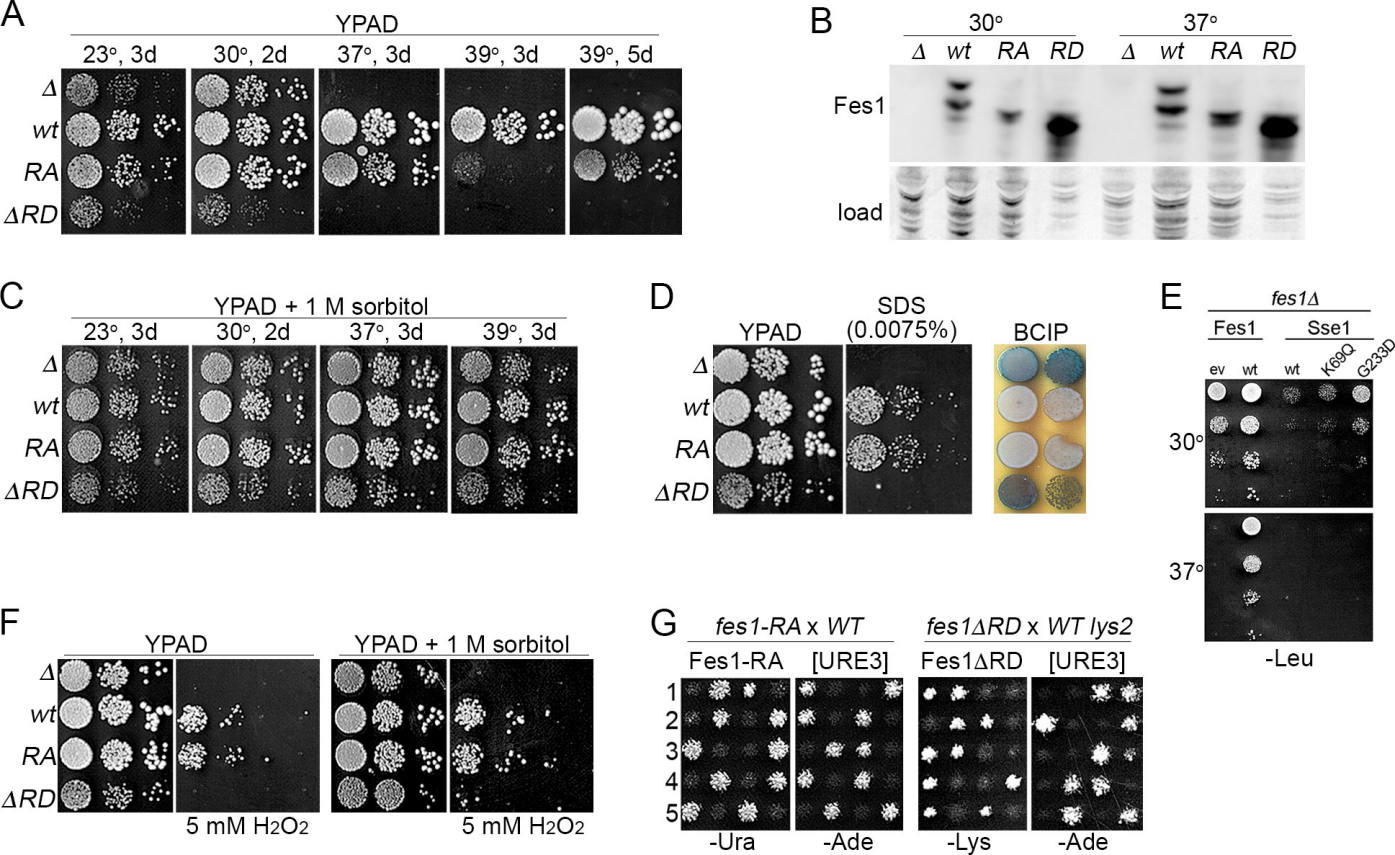
Fes1 NEF function, but not release domain, is dispensable for Fes1 functions in vivo. (A) Serial dilutions of strains lacking Fes1 (SY346, *Δ*) or expressing chromosomally encoded Fes1 wild type (1075, *wt*), Fes1^A79R,R195A^ (SKY207, *RA*) or Fes1ΔRD (1853–35, *ΔRD*) were plated on YPAD and grown as indicated. Each image is a single plate and the same cultures are on all plates. (B) Western analysis of Fes1 proteins from the same four strains in panel (A), as indicated, grown in liquid YPAD at the indicated temperature. For Fes1ΔRD lysates, 1/5 as much total protein was loaded on gels. Fes1 is expressed in both long (L) and short (S) forms, but the genomically expressed RA mutant is expressed only as the short form (see text). Image labeled "load" shows the blotted membrane stained by amido-black as loading and transfer controls. (C) As in panel (A), except plates contain 1 M sorbitol. (D) Left and center images show the same diluted strains plated in panel (A) grown 2 days at 30°C on YPAD without or with 0.0075% SDS as indicated. Image on right shows same strains grown 2 days on YPAD at 30°C and then overlaid with agar containing BCIP to detect leakage of alkaline phosphatase, which is seen as blue coloration. (E) Serial dilutions of *fes1Δ* cells with plasmids encoding Fes1 or indicated Sse1 proteins were plated on medium selecting for plasmid maintenance and grown at indicated temperatures for two days. Sse1-K69M is ATPase dead, but binds ATP and retains Hsp70 interaction and NEF function. Sse1-G233D does not bind ATP or act as a NEF {Shaner, 2004 #67}. Expressing Sse1 inhibits growth, but less so when its NEF activity is disrupted. (F) As in panel (A) except plates contain 5 mM H2O2 and 1 M sorbitol as indicated. (G) Five tetrads (1–5) each from sporulated diploids of parents indicated above were replica-plated onto -Ade medium, which selects for cells propagating [URE3] (see text), and medium selecting for cells expressing indicated *FES1* allele. The *fes1*^*A79R*,*R195A*^ strain (SKY207) is Ura^+^ (see Methods) and *fes1ΔRD* segregates with Lys^+^ (*FES1* is near *LYS2* on chromosome 2).

The earlier observations that temperature sensitivity of *fes1Δ* cells can be rescued by plasmid-based expression of wild type Fes1, but not by Fes1^A79R,R195A^, led to the conclusion that Fes1 NEF activity was essential for Fes1 function in vivo [14,16]. As with the earlier strain, growth of our *fes1Δ* mutant was near normal at optimal temperature (30°C), but severely compromised at 37°C (Fig 4A, Table 2). We found Fes1 also was important for growth at the sub-optimal 23°C. However, although the rate of growth of our *fes1*^*A79R*,*R195A*^ cells at 37°C was three times slower than wild type cells, they grew three times faster than the *fes1Δ* mutant (Table 2). When grown on plates at 37°C, *fes1Δ* cells did not form colonies at all, while the difference in growth between *fes1*^*A79R*,*R195A*^ and wild type cells was apparent, but much more subtle. Additionally, unlike cells lacking Fes1, those expressing Fes1^A79R,R195A^ were viable at 39°C, which is the upper limit for growth of most *S*. *cerevisiae* strains. They grew much more slowly at 39°C than wild type cells, which reflects reduced Fes1 activity, but they still formed colonies upon extended incubation (Fig 4A). Together these results show that Fes1^A79R,R195A^ retains substantial Fes1 function in vivo.

**Table 2:** Growth rates (min/cell div) of Fes1 mutant strains.

Temp.	wt	*fes1Δ*	*fes1*^*A79R*,*R195A*^	ΔRD
30°C	111 ± 6	116 ± 5	107 ± 4	170± 5
37°C	113 ± 4	1105 ± 196	358 ± 6	ND

Values are averages (± SD) from at least three independent cultures of each strain grown in liquid YPAD medium. ND, not determined; cells were inviable (>2000 min/cell div) under these conditions.

The *fes1ΔRD* cells failed to grow at 37°C, which agrees with earlier work [17], and they grew noticeably more slowly than *fes1Δ* cells at optimal temperature (Fig 4A, Table 2). These results indicate that interaction of Fes1 with Hsp70 is not enough to provide Fes1 functions needed for growth at non-optimal temperature and suggest that expression of Fes1ΔRD is toxic.

To determine if growth differences of these strains could be due to differences in expression, we compared steady-state abundance of the Fes1 proteins (Fig 4B). Fes1 encodes a longer splice variant (Fes1L) containing a C-terminal nuclear localization signal that is not needed for high temperature growth and other Fes1 functions [29]. Our chromosomal *fes1*^*A79R*,*R195A*^ allele does not produce this long form because it has *URA3* inserted just after the termination codon of Fes1. Fes1^A79R,R195A^ was a bit less abundant than that of wild type Fes1 and, as expected, the Fes1L version of Fes1^A79R,R195A^ was absent. Fes1ΔRD was expressed at levels much higher than wild type Fes1. Thus, reductions in growth are not explained simply by reductions in expression.

To try and resolve differences in Fes1^A79R,R195A^ phenotypes we see with those of the earlier work, we repeated those earlier experiments using the same strain and plasmid-based expression of Fes1 and Fes1^A79R,R195A^ regulated by the weak *ADH1* promoter [14,30,31]. As controls we included our *fes1Δ* strain and we repeated the experiments in both strains with alleles regulated by the *FES1* promoter that is activated by stress. The *FES1* promoter improved complementation by Fes1^A79R,R195A^, but not as effectively as integrating the *fes1*^*A79R*,*R195A*^ allele at the native *FES1* chromosomal locus (S2A–S2C Fig). Here again the results are consistent with Fes1^A79R,R195A^ functioning less well than wild type Fes1, but retaining substantial Fes1 activity. Apparently, regulation of Fes1 expression in its native context is important for its functions in vivo, which could be related to maintaining a balance of interacting factors whose expression is co-induced by the same environmental conditions.

Temperature sensitivity that is associated with defects in cell wall integrity (CWI) can be suppressed by osmotic support in the growth medium. We added 1M sorbitol to the medium used for growth assays to provide such support and found growth of *fes1Δ* and *fes1ΔRD* cells was restored even at 39°C (Fig 4C). We tested our strains for other characteristics of cell wall defects and found *fes1Δ* and *fes1ΔRD* cells were hypersensitive to SDS and they leak alkaline phosphatase, even at 30°C where a growth defect is not so pronounced (Fig 4D) [32,33]. Cells expressing Fes1^A79R,R195A^ showed no indication of SDS hypersensitivity or cell wall leakage at 30°C, suggesting the role of Fes1 in CWI does not require NEF function. Moreover, the temperature sensitivity of *fes1Δ* cells was not suppressed by elevating expression of Sse1 (Fig 4E), which is consistent with the CWI defect of *fes1Δ* cells not being due simply to a loss of NEF function. Thus, the loss of Fes1 causes several phenotypes diagnostic of CWI defects that can be overcome by Fes1^A79R,R195A^, which lacks Hsp70-binding and NEF function, but not by Fes1ΔRD.

As it is unlikely that sorbitol helps Hsp70 release substrates, we suspect the CWI defect in *fes1Δ* cells is related to loss of a Fes1 activity that is retained by Fes1^A79R,R195A^ rather than the loss of Hsp70 NEF activity. Accordingly, we found that while *fes1Δ* and *fes1ΔRD* cells were hypersensitive to hydrogen peroxide, which is an oxidative stress not specific to cell wall damage, Fes1^A79R,R195A^ also suppressed this sensitivity, but 1M sorbitol did not (Fig 4F). Thus, sorbitol is not a general suppressor of phenotypes caused by lack of Fes1, and Fes1 can perform PQC functions important for growth under stress beyond CWI that do not require its NEF regulation of Hsp70, but do require its RD.

[URE3] prions are composed of self-assembling amyloid aggregates of the transcriptional regulator Ure2 [34,35]. The replication of these aggregates that is necessary for their continued distribution among dividing cells depends on their fragmentation by the protein disaggregation machinery composed of Hsp104, Hsp70, Hsp40 and NEF [36]. Fes1 is required for propagation of [URE3] prions [37,38] and this dependence provides another measure of Fes1 function in vivo.

We monitor [URE3] in our strains using *ADE2* regulated by the *DAL5* promoter, which is repressed by Ure2 so cells are Ade^–^. When [URE3] is present, Ure2 is depleted into amyloid aggregates and cannot maintain this repression so cells are Ade^+^. To test if Fes1^A79R,R195A^ or Fes1ΔRD could promote prion propagation, we monitored [URE3] among meiotic progeny of [URE3] diploids heterozygous for wild type *FES1* and *fes1*^*A79R*,*R195A*^ or *fes1ΔRD* (Fig 4G). Among progeny of 20 tetrads for each diploid, all those expressing wild type Fes1 propagated [URE3] stably, while all of those expressing Fes1^A79R,R195A^ or Fes1ΔRD did not. Thus, neither Fes1^A79R,R195A^ nor Fes1ΔRD supported [URE3] propagation. These results agree with the conclusion made by others that these Fes1 mutants do not cooperate with Hsp70 in vivo. They also align with the requirement that all other Hsp70 co-chaperones known to be important for [URE3] propagation, including NEFs Snl1 and Sse1, interact functionally with Hsp70 [16,37,39–42].

### Fes1 interacts with cell wall integrity MAPK signaling kinase Slt2

**Fig 5:**
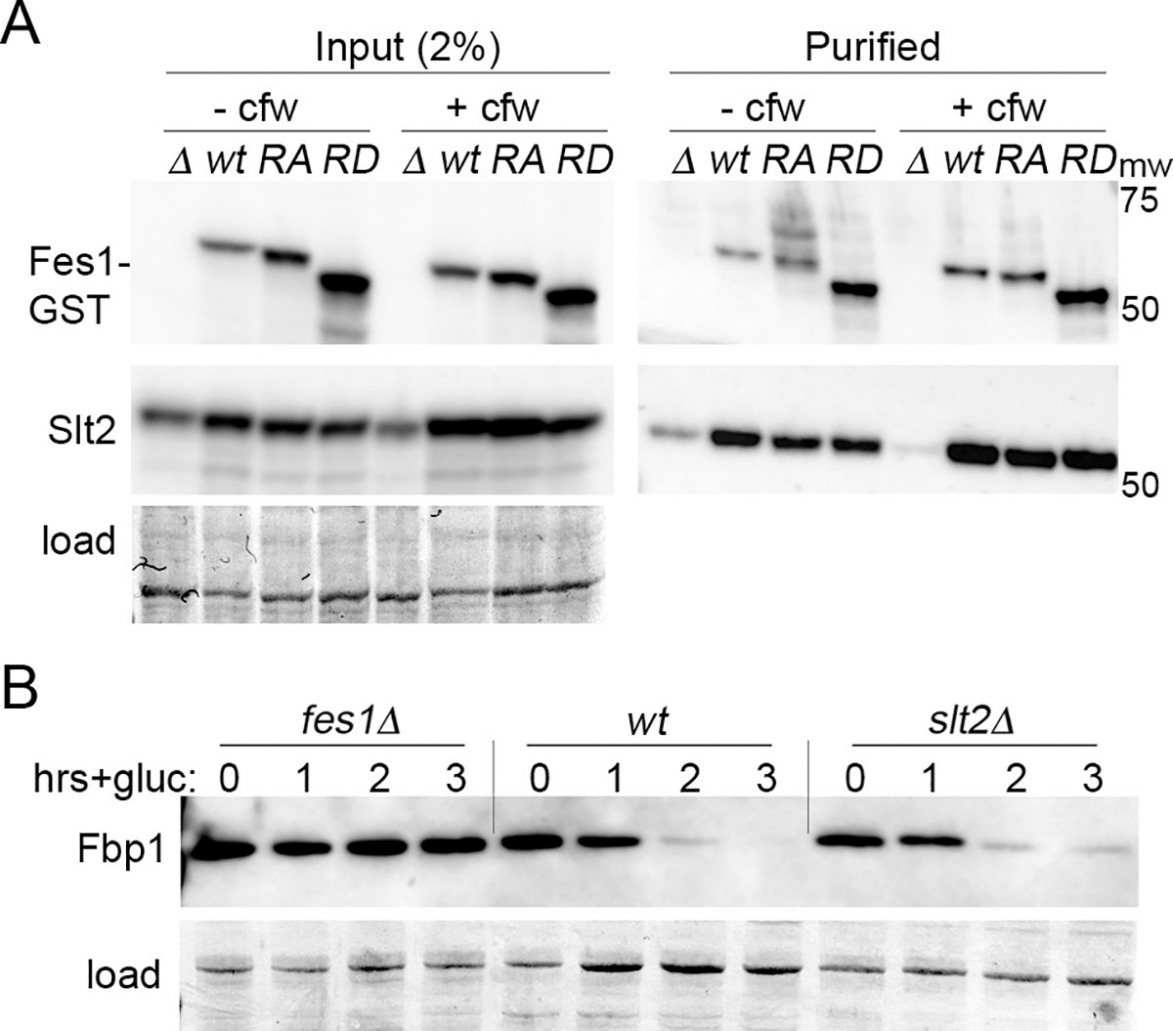
Fes1 and Fes1 mutants interact with CWI kinase Slt2. All panels show western analysis of indicated protein. (A) Fes1-GST was purified from lysates of *fes1Δ* cells expressing plasmid-encoded wild type Fes1 (*wt*), Fes1^A79R,R195A^ (*RA*) or Fes1ΔRD (*RD*) grown at 30°C with (+cfw) or without (-cfw) calcofluor white treatment for 60 min. Left (Input), whole lysates blotted and probed for Fes1 and Slt2. Right (Purified), GST purified proteins processed similarly. (B) Vid assay, as in Fig 1A, shows Vid is functional in cells lacking Slt2. Images labeled "load" show blotted membranes stained by amido-black as loading and transfer controls.

Cell wall stress activates a mitogen-activated protein kinase (MAPK) signaling pathway that is controlled by MAP kinase Slt2 [43,44]. Activation of Slt2 depends on the Hsp90/Hsp70 chaperone system and this pathway can be activated 2-3-fold in cells lacking Fes1 [45,46]. In light of our findings that Fes1 is essential for Vid and can bind Fbp1, we tested if the relationship between Fes1 and defective cell walls might involve an interaction between Fes1 and Slt2. We purified Fes1 from lysates of *fes1Δ* cells that express wild type or mutant versions of Fes1-GST from plasmids and looked for co-purification of Slt2 (Fig 5A). We also repeated these experiments using the same cultures of cells treated with the cell wall-specific stressor calcofluor white. Slt2 co-purified with wild type Fes1, Fes1^A79R,R195A^ and Fes1ΔRD from lysates of both treated and untreated cells. For all strains, more Slt2 co-purified from cells exposed to calcofluor white, which corresponded to an increased amount of Slt2 in the treated strains. Thus, Fes1 and Slt2 interact in vivo and the ΔRD or combined A79R and R195A mutations do not disrupt this interaction.

We were unable to purify Slt2 in a soluble form, so we cannot confirm whether this interaction could be direct. Nevertheless, together with our other data these results suggest that this interaction is important for CWI signaling, that it does not require Hsp70 binding or NEF function of Fes1, that the armadillo domain mediates the interaction, and that the functional output of the interaction requires the RD. We further found Vid degradation of Fbp1 was normal in cells lacking Slt2 (Fig 5B), indicating that Slt2 is not important for Vid and that any interaction between Fes1 and Slt2 is unrelated to this protein degradation pathway. Our findings that the RD is needed for CWI, but not Vid, are in line with Fes1 acting differently in these two processes.

### RD of Fes1 is needed for cell wall integrity

**Fig 6:**
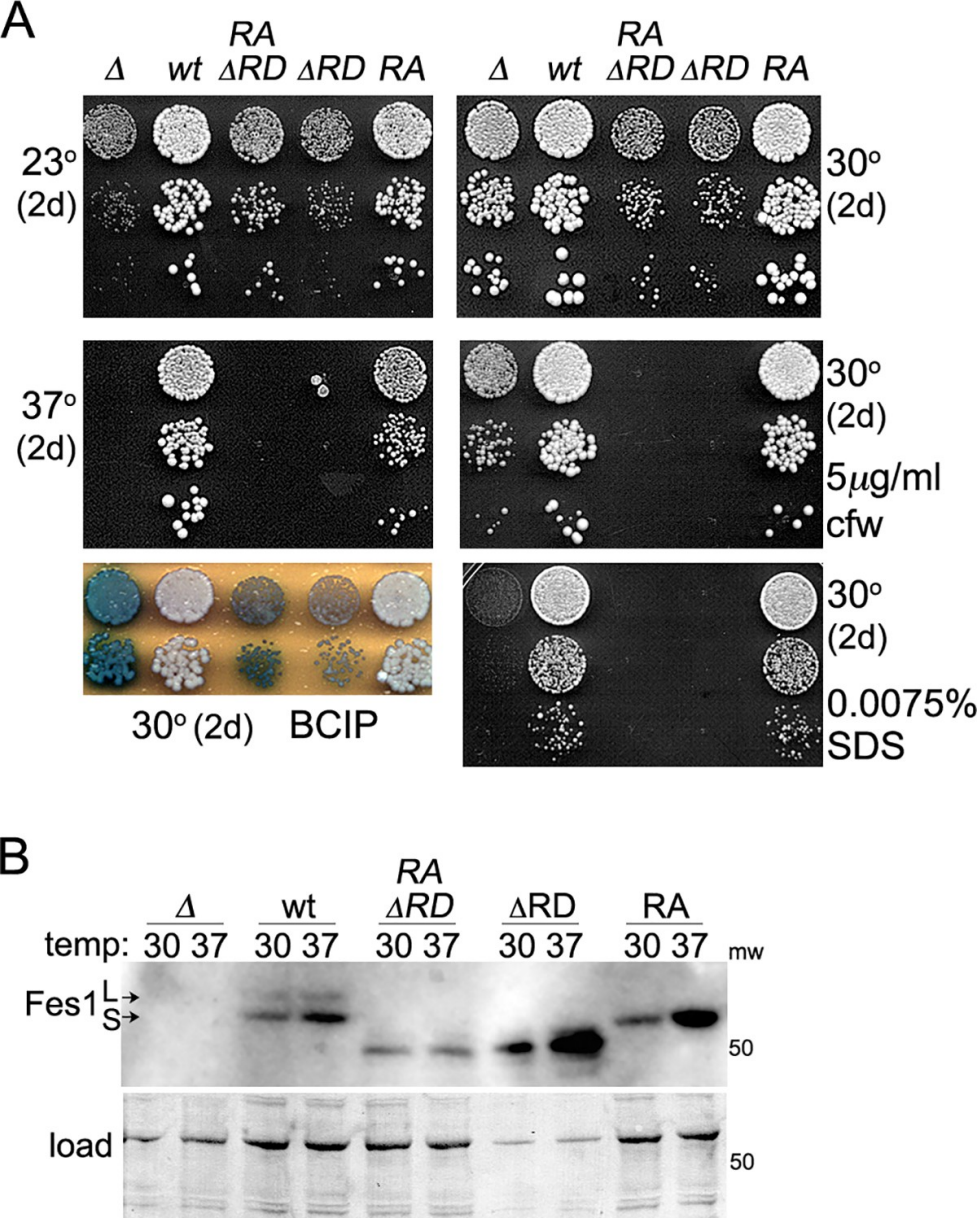
Fes1^A79R,R195A^ΔRD behaves more like Fes1ΔRD than Fes1^A79R,R195A^. (A) Cultures of cells lacking Fes1 (*Δ*) or those expressing wild type Fes1 (1075, *wt*), Fes1^A79R,R195A^ΔRD (1890, *RAΔRD*), Fes1ΔRD (1853–35, *ΔRD*) or Fes1^A79R,R195A^ (SKY207, *RA*) were diluted and plated as in Fig 4A. (B) Western blot analysis to compare abundance of Fes1 from lysates of strains used in panel (A) that were grown in YPAD liquid at 30°C or 37°C for 5 hrs before collecting cells for analysis. For Fes1ΔRD lysates, 1/5 as much total protein was loaded on gels.

Wild type Fes1 and Fes1^A79R,R195A^ maintained cell wall integrity, but Fes1ΔRD did not, which suggest that the RD has a function in CWI unrelated to its role in regulating Hsp70. If so, then deleting the RD from Fes1^A79R,R195A^ (creating Fes1^A79R,R195A^ΔRD) should impair ability of Fes1^A79R,R195A^ to support CWI. Alternatively, if the phenotypes of cells expressing Fes1ΔRD are due to impairment of Hsp70 function by non-productive binding of Fes1ΔRD to Hsp70, then Fes1^A79R,R195A^ΔRD, which should not bind Hsp70, should support CWI like Fes1^A79R,R195A^. We found that a strain expressing Fes1^A79R,R195A^ΔRD in place of Fes1 from its genomic locus grew somewhat faster than *fes1Δ* or *fes1ΔRD* mutants at 23°C (Fig 6A). Otherwise, it grew more slowly than *fes1Δ* cells at optimal temperature (30°C), was more sensitive to high temperature and to the cell wall stressors SDS and calcofluor white, and leaked alkaline phosphatase at 30°C. These phenotypes resemble those of cells expressing Fes1ΔRD rather than those expressing Fes1^A79R,R195A^, which shows growth under these conditions depends on a function of the RD and that the RD has a role in CWI beyond its acting to prevent rebinding of substrates released by Hsp70. Not surprisingly, Fes1^A79R,R195A^ΔRD did not support propagation of [URE3], as seen for both Fes1^A79R,R195A^ and Fes1ΔRD (S3 Fig).

At 30°C the Fes1^A79R,R195A^ΔRD protein was expressed at a level much lower than that of Fes1ΔRD (Fig 6B), which suggests that negative effects on growth caused by expressing only the armadillo domain of Fes1 do not require high expression or interaction with Hsp70. Additionally, unlike Fes1^A79R,R195A^, the abundance of Fes1^A79R,R195A^ΔRD was not increased after a shift to elevated temperature, which suggests the RD and an interaction of Fes1 with Hsp70 can combine to influence Fes1 stability or expression.

## Discussion

Although all functions of Fes1 are thought to be mediated by its role in helping Hsp70 release ADP and substrates, we find that Fes1 mutants lacking these activities retain important functions in different cellular processes, which shows that Fes1 performs functions that are separate from its regulation of Hsp70. We show Fes1 interacts with proteins involved in these processes, which reveals a non-Hsp70 protein-binding activity of Fes1 and suggests Fes1 could act in its roles by binding to these proteins.

Although Fes1 is not needed for proteasomal degradation of Fbp1 after short-term starvation, we find Fes1 is needed for efficient degradation of Fbp1 when cells undergo prolonged starvation. Thus, Fes1 is not required for cells to switch away from the proteasome pathway, but it is needed for Vid after cells have committed to the switch. The specific role of Fes1 in Vid remains to be determined, but our results showing Fes1 binds similarly to Ssa1 and Ssa2 and that Fes1^A79R,R195A^ and Fes1ΔRD supported Vid function imply that this role does not involve Fes1 regulation of Ssa2 or underlie the functional differences between Ssa1 and Ssa2. That Fes1^A79R,R195A^ and Fes1ΔRD also retain ability to bind Fbp1 suggests the role of Fes1 in Vid could be linked to this interaction and we suspect Fes1 might bind other Vid substrates.

Our data do not rule out the formal possibility that Fes1^A79R,R195A^ retains ability to bind and regulate Hsp70 in cells at some obviously reduced level. However, ample evidence provided here and by others implies that Fes1^A79R,R195A^ does not function as a NEF for Hsp70 in a physiologically meaningful way. Based on crystal structure data of human Fes1 homolog HspBP1 bound to Hsp70, the A79R and R195A mutations in Fes1 were designed to disrupt Hsp70 binding and then shown to have that effect on binding Ssa1 in vitro and in vivo [14,16,28,29]. We confirmed the in vitro findings and extended them to Ssa2, which is more abundant in vivo. We further found Fes1^A79R,R195A^ did not support propagation of [URE3], which exemplifies the expected loss of Hsp70-NEF activity.

We see differences in phenotypes of Fes1^A79R,R195A^ with those reported earlier that apparently are due to differences in gene expression and growth conditions used. As in the earlier work [16,17], we find plasmid-based expression of Fes1^A79R,R195A^ gave generally reduced (and variable) complementation, in particular when using the weak *ADH1* promoter. Integrating alleles into native chromosomal loci avoids uncontrollable variations in expression and resulted in the strongest complementation. In agreement with earlier data, we do see considerably reduced ability of Fes1^A79R,R195A^ to support growth at the extreme temperature of 39°C, which clearly exposes functional deficiencies of this mutant. At the universally applied stringent temperature of 37°C, however, growth differences of wild type and *fes1*^*A79R*,*R195A*^ cells were much less notable.

Additionally, whereas depleting Fes1 abolished Vid function and caused readily identifiable growth defects under other stress conditions, cells expressing Fes1^A79R,R195A^ natively from its chromosomal locus were for the most part phenotypically similar to wild type cells. Thus, loss of Fes1 NEF function is much less physiologically detrimental than loss of Fes1, which implies that *fes1Δ* cells suffer from something greater than loss of Fes1 NEF activity. Accordingly, the temperature sensitivity of *fes1Δ* cells is not suppressed by elevating expression of the Hsp70 NEF Sse1, which also is important for cell wall integrity [33]. In contrast, the CWI defect of *sse1Δ* cells is due to reduced NEF activity that can be suppressed by elevating Fes1 [33]. Thus, Fes1 contributes to CWI in a way that Sse1 does not and the need of Fes1 for CWI can be accomplished by a non-NEF function. Overall our data indicate Fes1^A79R,R195A^ retains Fes1 functions that are important for cellular fitness.

In contrast, cells expressing Fes1ΔRD, which binds Hsp70 and retains NEF function, had normal Vid function, but otherwise were even less fit than cells lacking Fes1. It was proposed that growth defects associated with inability of Fes1ΔRD to ensure release of substrates from Hsp70 are caused by persistent binding of proteasome-targeted substrates to Hsp70, which reduces both availability of Hsp70 and delivery of the substrates to proteasomes [17]. The conclusion that Fes1ΔRD is not only defective, but also could interfere with cellular processes that depend on Hsp70 by binding Hsp70 non-productively is in line with its high expression and the growth defects of *fes1ΔRD* cells being more pronounced than those of *fes1Δ* cells. Our findings that Fes1 ^A79R,R195A^ΔRD was expressed at much lower abundance and still behaved like Fes1ΔRD, however, indicate that negative effects of Fes1ΔRD on growth do not require its elevated expression or interaction with Hsp70.

We emphasize, moreover, that sorbitol, an osmotic stabilizer that is a widely recognized suppressor of temperature sensitivity caused by cell wall defects, very effectively overcame sensitivity of both *fes1Δ* and *fes1ΔRD* cells to even extreme temperature. It is difficult to envision how the relatively inert sorbitol could overcome severe and classic CWI phenotypes specifically by helping Hsp70 release ADP or substrates, especially as we show sorbitol is not a general suppressor of Fes1 deficiency. We presume the major growth defects of Fes1 mutants are more likely due to loss of an Hsp70-independent function of Fes1 in cell wall integrity rather than to lost or damaged ability of Fes1 to regulate Hsp70.

Our findings establish an important role for Fes1 in cell wall integrity and imply this role does not require its NEF function. We suppose the interaction of Fes1 with Slt2 is likely an important part of this role. This interaction was detected in cell lysates, so we cannot rule out that it is indirect. Fes1^A79R,R195A^ also bound Slt2, however, implying that any indirect interaction does not occur through binding of Fes1 to Hsp70 in a ternary complex. Additionally, Fes1ΔRD bound Slt2, but failed to maintain CWI, showing that this interaction alone is not enough to maintain cell wall integrity. Together with our results showing Fes1^A79R,R195A^ΔRD is also defective in CWI, these findings suggest the RD contributes importantly to such a role whether or not Fes1 binds Hsp70.

In addition to its role in cell wall integrity, Slt2 (Erk5 in humans) acts in a separate conserved MAPK signaling pathway that regulates proteasome abundance [47,48]. Although proteasome defects in cells with depleted or mutated Fes1 are attributed to inability of Fes1 to facilitate release of substrates from Hsp70 to the proteasome, our data suggest that altered Slt2 signaling caused by mutating Fes1 could contribute to the reduced proteasome function of Fes1 mutants.

Fes1 is in the armadillo repeat family, which has members that can interact with multiple partners and have diverse functions [49]. It is not entirely surprising, then, to find that Fes1 can bind proteins other than Hsp70. That it does so even when carrying mutations that disrupt its binding to Hsp70 reveals a specificity in binding of Fes1 to unrelated proteins. Our pull-down data showing Fbp1 binds more Fes1^A79R,R195A^ than wild type Fes1 in reactions with Hsp70 (Table 1) suggest that binding of Fes1 to Hsp70 could reduce its availability to bind other proteins. Working out the details of how the binding of Fes1 to non-Hsp70 proteins is regulated, how it might influence functions of such proteins and whether non-NEF activities of Fes1 are evolutionarily conserved are intriguing areas for future work.

## Materials and methods

### Strains, plasmids, and growth conditions

Strains are listed in Table 3. Standard methods were used to construct strains with mutant alleles [50]. The *fes1*^*A79R*,*R195A*^ allele in strain SKY207 has *URA3* with its promoter inserted immediately after the termination codon. It was created by integrative transformation of strain 1075 with the allele excised from a plasmid and selecting for transformants on -Ura plates. The *fes1ΔRD* strain 1853–35 is identical to wild type strain 1075 except it lacks codons 2–34 of *FES1*. Strain 1890 is identical to strain 1853–35 except it also has the A79R and R195A mutations in *FES1*. Both were created by co-transforming a [URE3] version of strain 1075 using pRS316 and PCR products containing the mutant alleles and selecting transformants on -Ura medium. Ura^+^ transformants were then screened for loss of [URE3]. Presence of [URE3] was monitored by ability to grow on adenine (see text). Mutant strains generated by integrative transformation were verified by PCR, sequencing and western analysis. Except for strain 1890, expression of only the desired Fes1 variant was verified further by mass-spectrometry. Standard yeast media and growth conditions were used [50]. Cells were grown at 30°C unless indicated otherwise. Glucose-rich YPAD contains 1% yeast extract, 2% peptone, 0.04% adenine and 2% dextrose. Glucose-limiting YPKAG is the same except it contains 0.5% dextrose and 1% potassium acetate. Synthetic media contain 2% glucose, 7 gm/L Yeast Nitrogen Base (Difco) and complete supplement mix (Sunrise Science Products) lacking only nutrients needed to maintain selection of plasmids or prions.

**Table 3:** Strains used in this study.

Strain	Relevant genotype	Source
1075	*MAT****a*** *P*_*DAL5*_:*ADE2 kar1-1 SUQ5 his3 leu2 trp1 ura3*	[53]
SY135	*ssa1*::*KanMX ssa2*::*HIS3 ssa3*::*TRP1 ssa4*::*ura3-2f* /pRS315-A1	[10]
SY136	*ssa1*::*KanMX ssa2*::*HIS3 ssa3*::*TRP1 ssa4*::*ura3-2f* /pRS315-A2	[10]
SY346	*fes1*::*KanMX*	[10]
SY351	*snl1*::*KanMX*	This study
SKY207	*MATα fes1*^*A79R*,*R195A*^::*URA3*	This study
1853–35	*MATα fes1ΔRD*	This study
1890	*MAT**a** fes1*^*A79R*,*R195A*^*ΔRD*	This study
BY4741slt2	*MAT****a*** *slt2*::*KanMX his3 leu2 met15 ura3*	Euroscarf
BY241ΔSSE	*MAT****a*** *kar1 leu2 sse1*::*KanMX trp1 ura3 P*_*DAL5*_::*ADE2 P*_*DAL5*_::*CAN1*	[37]
1821	(YPH499) *MAT**a** ade2-101 his3 leu2 lys2 trp1 ura3*	[54]
1822	1821 *fes1*::*KanMX*	This study

All are isogenic to 1075 except BY4741slt2, BY241ΔSSE, 1821, and 1822.

Plasmids used are described in Table 4. All plasmids generated in this study were constructed using standard recombinant DNA methods. Plasmid p315FES1 contains *FES1* coding region with 437 bp of 5' and 256 bp of 3' flanking DNA. Plasmids with mutant versions of Fes1 are identical except where indicated in the *FES1* coding region.

**Table 4:** Plasmids used in this study.

Plasmid	Description	Source
pRS425	2μ, LEU2 (multi-copy)	[55]
p415	*CEN*, *LEU2* (single-copy)	[30]
pRS314	*CEN*, *TRP1* (single-copy)	[54]
pRS315	*CEN*, *LEU2* (single-copy)	[54]
p315FES1	pRS315, *P*_*FES1*_::*FES1*	This study
p315FES1RA	pRS315, *P*_*FES1*_::*fes1*^*A79R*,*R195A*^	This study
p315FES1ΔRD	pRS315, *P*_*FES1*_::*fes1Δ2–34*	This study
p315FES1-GST	pRS315, *P*_*FES1*_::*FES1-GST*	This study
p315FES1RA-GST	pRS315, *P*_*FES1*_:: *fes1*^*A79R*,*R195A*^*-GST*	This study
p315FES1ΔRD-GST	pRS315, *P*_*FES1*_::*fes1*Δ*RD-GST*	This study
p313-GST	pRS313, *P*_*FES1*_::*GST*	This study
p415ADH	p415, *P*_*ADH1*_	This study
p415ADHFES1	p415, *P*_*ADH1*_::*FES1*	This study
p415ADHFES1RA	p415, *P*_*ADH1*_::*fes1*^*A79R*,*R195A*^	This study
pET28aFBP1	*E*. *coli* expression, N-His6 tag	This study
pET28aFBP1P2A	*E*. *coli* expression, N-His 6tag	This study
pET28aFBP1P2Δ	*E*. *coli* expression, N-His6 tag	This study
pET28aFBP1-P2AS12AT13A	*E*. *coli* expression, N-His6 tag	This study
pET28aFBPΔ2–12	*E*. *coli* expression, N-His6 tag	This study
pET24-SSA1	*E*. *coli* expression, No tag	[51]
pMR299 (Ssa2)	*E*. *coli* expression, No tag	[52]
pGEX5AFES1	*E*. *coli* expression, N-GST tag	[15]
pGEX5AFES1RA	*E*. *coli* expression, N-GST tag	This study
pGEX5AFES1ΔRD	*E*. *coli* expression, N-GST tag	This study
pRS425SSE1	*SSE1* wild type	[37]
pRS425SSE1K69Q	*sse1K69Q* mutant	[37]
pRS425SSE1G233D	*sse1G233D* mutant	[37]

### Protein expression and purification

Non-tagged Ssa1 and Ssa2 were purified from *E*. *coli* Rosetta 2(DE3) as described [51]. GST-Fes1 was purified as described [15]. His6-Fbp1 was expressed in Rosetta 2(DE3) pLysS and purified by standard metal affinity methods. Proline at position 2 of Fbp1 is often referred to as amino acid residue P1 because the initiator methionine of Fbp1 is removed. We refer to it as P2 because the methionine is present in purified Fbp1 and to avoid confusion regarding names of Fbp1 with N-terminal deletions.

### Vid assay

Strains grown in YPKAG for 3 days at 30°C were shifted to YPAD. Samples were collected immediately and 1 h, 2 h and 3 h post shift and treated immediately with 10 mM sodium azide. Cells were washed with water, suspended in lysis buffer (50 mM Tris.HCl-7.5, 150 mM NaCl, 0.1% Triton-X100 and proteinase inhibitors) and lysed by agitation with glass beads. Proteins (20 μg) were separated on 12% SDS-PAGE gels and transferred to PVDF membranes, which were processed by standard immunoblotting techniques using antibodies against Fbp1.

### In vitro pull-downs (PD)

Six μg each of His6-Fbp1 (1.6 μM), Hsp70 (Ssa1 or Ssa2, each 0.9 μM) and GST-Fes1 (1.09 μM) were incubated with or without ATP, ADP or AMP (2.5 mM) in buffer PD (50 mM Tris-HCl pH 7.5, 150 mM NaCl, 2% glycerol, 5 mM MgCl_2_) for 30 min at 4°C. His6-Fbp1 was purified using Talon resin pre-equilibrated with buffer PD. Resin was washed twice with wash buffer (PD buffer + 5 mM imidazole) and proteins were eluted in PD buffer containing 250 mM imidazole. Pull-downs using GST-Fes1 were performed as described [15]. Proteins were separated on 12% SDS-PAGE gels and stained with PageBlue (ThermoScientific, cat. no. 24620). All variants of Fbp1 have N-terminal His6 tags and all variants of Fes1 have N-terminal GST tags.

#### In vivo pull-downs and western analysis

Pull downs Fes1 and its mutants were done as described [52]. Briefly, *fes1Δ* strain SY346 expressing Fes1-GST fusion constructs (GST fused to Fes1 C-terminus) from a plasmid were grown at 30°C with or without calcofluor white, harvested, lysed by agitation with glass beads and debris was cleared by centrifugation. GST-fused proteins were purified by incubating lysates with GST-beads, washing the resin with buffer PD and eluting with buffer PD containing 20 mM glutathione.

For western analysis, lysates and purified proteins were separated on 12% SDS-PAGE gels, blotted using standard methods and probed using antibody against Fes1 (this study) or Slt2 (Santa Cruz Biotechnology; mouse monoclonal antibody MPK1 (E-8): sc374440). For cells expressing chromosomally encoded versions of Fes1, cells were grown overnight in YPAD, diluted 10-fold and grown an additional 5 hr at 30°C or 37°C. Except for samples from Fes1ΔRD lysate that contain 3 μg of total protein, lysate samples contained 15 μg of total protein. Analysis of plasmid-expressed versions of Fes1 in *fes1Δ* cells was done simlarly except cells were first grown in -Leu selective medium.

### Conditional growth tests and growth rates

Cells grown overnight in YPAD were diluted in fresh medium to OD_600_ = 0.1, grown to OD_600_ = 0.7 and diluted to OD_600_ = 0.2. Eight μl of a 10-fold dilution series was plated on YPAD supplemented and incubated as indicated. Colony sizes qualitatively reflect rates of growth. Growth rates were quantified by diluting overnight cultures to OD_600_ = 0.05 in 24 well plates (Corning Costar) and incubated with continuous shaking at 30°C or 37°C for 24 hr on an automated plate reader (SPECTROstar Omega, BMG labtech) with readings taken at 10 min intervals.

### BCIP assay

Cell wall defects were assessed by using BCIP as described [33]. Briefly, cells were diluted to OD_600_ = 0.02 and 8 μl of this and further 10-fold dilutions were plated on YPAD. Plates were incubated as indicated, overlaid with 5 ml of 1% agar containing 10 mM 5-bromo-4-chloro-3-indolyl phosphate (BCIP, Sigma cat. no B6149) in 0.05 M glycine buffer (pH 9.5) and then incubated at room temperature for up to 2 hr.

## Supporting information

S1 Fig Fbp1 amino acids 2–12 do not contribute to Fes1 binding. All panels show Coomassie-stained SDS-PAGE gels where protein pulled down is indicated on top rows. (A) His6-Fbp1ΔP2 was pulled down using metal affinity. Deleting residue P2 has no noticeable effect on binding to Ssa1 (lanes 1–2), Ssa2 (lanes 3–4) or Fes1 (lanes 6–7; compare all with Fig 2B). (B) As in panel (A) except His6-Fbp1^P2A,S12A,T13A^ was pulled down. Combined mutations have no effect on binding to Ssa1 (lanes 1–3), Ssa2 (lanes 4–6) or Fes1 (lanes 1–8). (C) As in panel (A) except His6-Fbp1Δ2–12 (lanes 1–3 and 7–19) or wild type His6-Fbp1 (lanes 4–6 and 20–22) was pulled down. (D) As in panel (A) except GST-Fes1 was pulled down using glutathione resin and additional reactions containing different combinations of proteins and nucleotides were included. Asterisks in panels (C) and (D) indicate position of contaminant sometimes found in Fbp1 preparations.
(TIF)

S2 Fig Fes1^A79R,R195A^ supports growth better when regulated by its native promoter. (A) Plasmid transformants of strain 1822 (YPH499 *fes1Δ*) with empty vector (ev) or the vector encoding Fes1 (wt) or Fes1^A79R,R195A^ (RA) were grown in -Leu liquid medium, diluted, plated on -Leu or YPAD and incubated at indicated temperature for 2–3 days as indicated. Cells express Fes1 from the weak *ADH1* promoter [14,30,31] or the native *FES1* promoter as indicated (prom). When Fes1^A79R,R195A^ is regulated by the *FES1* promoter growth at 37°C is more noticeable. (B) As in (A) except using our *fes1Δ* strain SY346 and plasmids expressing Fes1ΔRD were included. (C) Western analysis of Fes1 proteins from the same strains as in panel (A) (lanes 1–12) and panel (B) (lanes 13–17), as indicated, grown in liquid YPAD at the indicated temperature. Image labeled "load" shows the blotted membranes stained by amido-black as loading and transfer controls. Growth differences do not seem to be due simply to differences in expression.
(TIF)

S3 Fig Fes1^A79R,R195A^ΔRD does not support normal cell growth or [URE3] propagation. Dissected tetrads of sporulated [URE3] diploids of parents indicated above were replica-plated onto -Lys medium selecting for cells expressing Fes1^A79R,R195A^ΔRD (*FES1* is linked to *LYS2* on chromosome 2) and medium lacking adenine (-Ade), which selects for cells propagating [URE3] (see text). Five of the tetrads shown (1–5) have four viable spores. On primary dissection plate (YPAD) all Fes1^A79R,R195A^ΔRD colonies are smaller than wild type colonies, which implies the slower growth caused by expression of Fes1 lacking its RD is not due to a growth-inhibitory interaction of Fes1ΔRD with Hsp70.
(TIF)
