# Supplementary figures and images for: Hsp70-nucleotide exchange factor (NEF) Fes1 has non-NEF roles in degradation of gluconeogenic enzymes and cell wall integrity
Source: PLoS Genet. 2019 Jun 26;15(6):e1008219. doi: 10.1371/journal.pgen.1008219 (PMC6615629; doi:10.1371/journal.pgen.1008219)

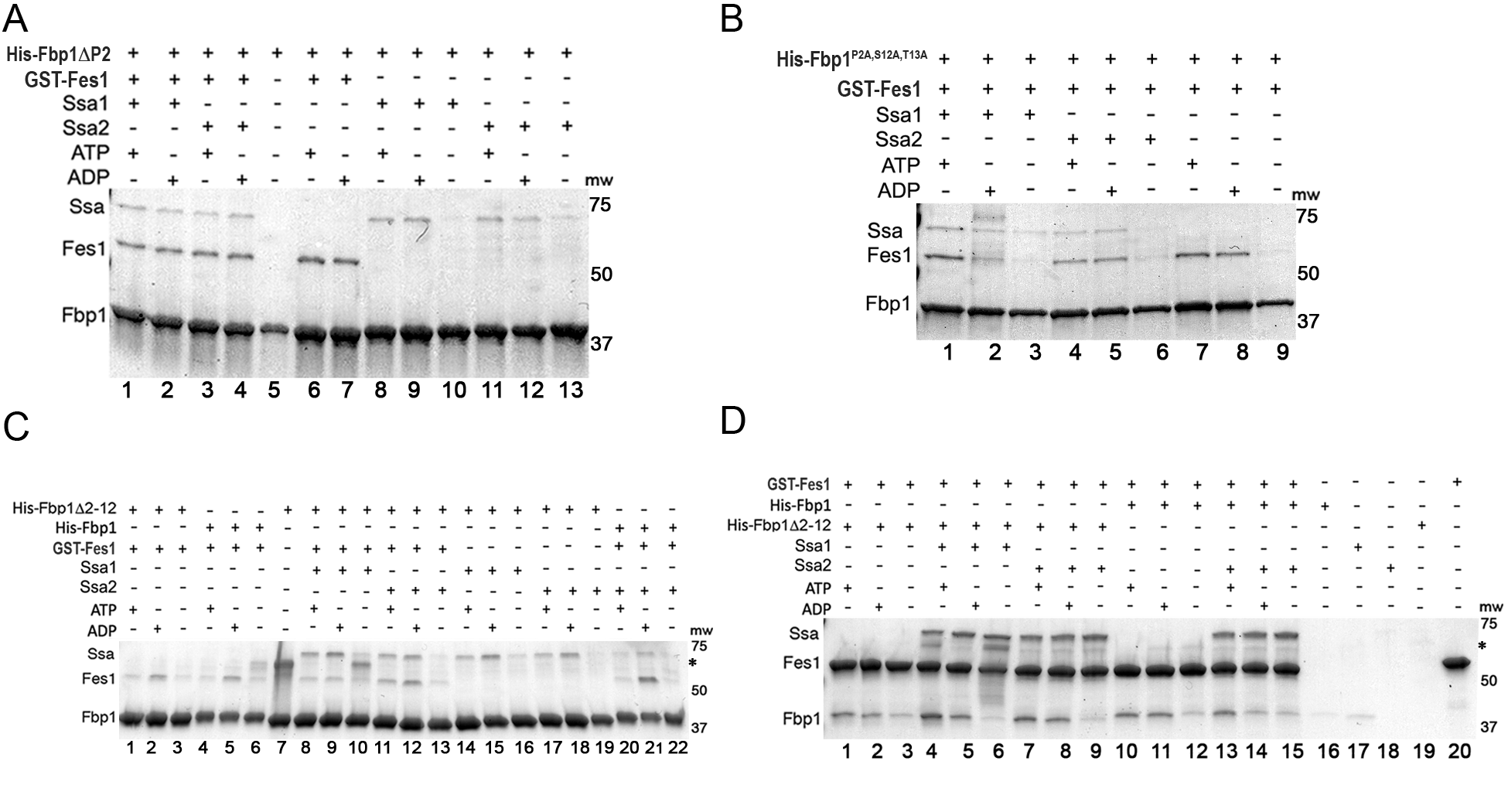

Supplement: S1 Fig — All panels show Coomassie-stained SDS-PAGE gels where protein pulled down is indicated on top rows. (A) His6-Fbp1ΔP2 was pulled down using metal affinity. Deleting residue P2 has no noticeable effect on binding to Ssa1 (lanes 1–2), Ssa2 (lanes 3–4) or Fes1 (lanes 6–7; compare all with Fig 2B). (B) As in panel (A) except His6-Fbp1P2A,S12A,T13A was pulled down. Combined mutations have no effect on binding to Ssa1 (lanes 1–3), Ssa2 (lanes 4–6) or Fes1 (lanes 1–8). (C) As in panel (A) except His6-Fbp1Δ2–12 (lanes 1–3 and 7–19) or wild type His6-Fbp1 (lanes 4–6 and 20–22) was pulled down. (D) As in panel (A) except GST-Fes1 was pulled down using glutathione resin and additional reactions containing different combinations of proteins and nucleotides were included. Asterisks in panels (C) and (D) indicate position of contaminant sometimes found in Fbp1 preparations. (TIF) [file pgen.1008219.s001.tif]

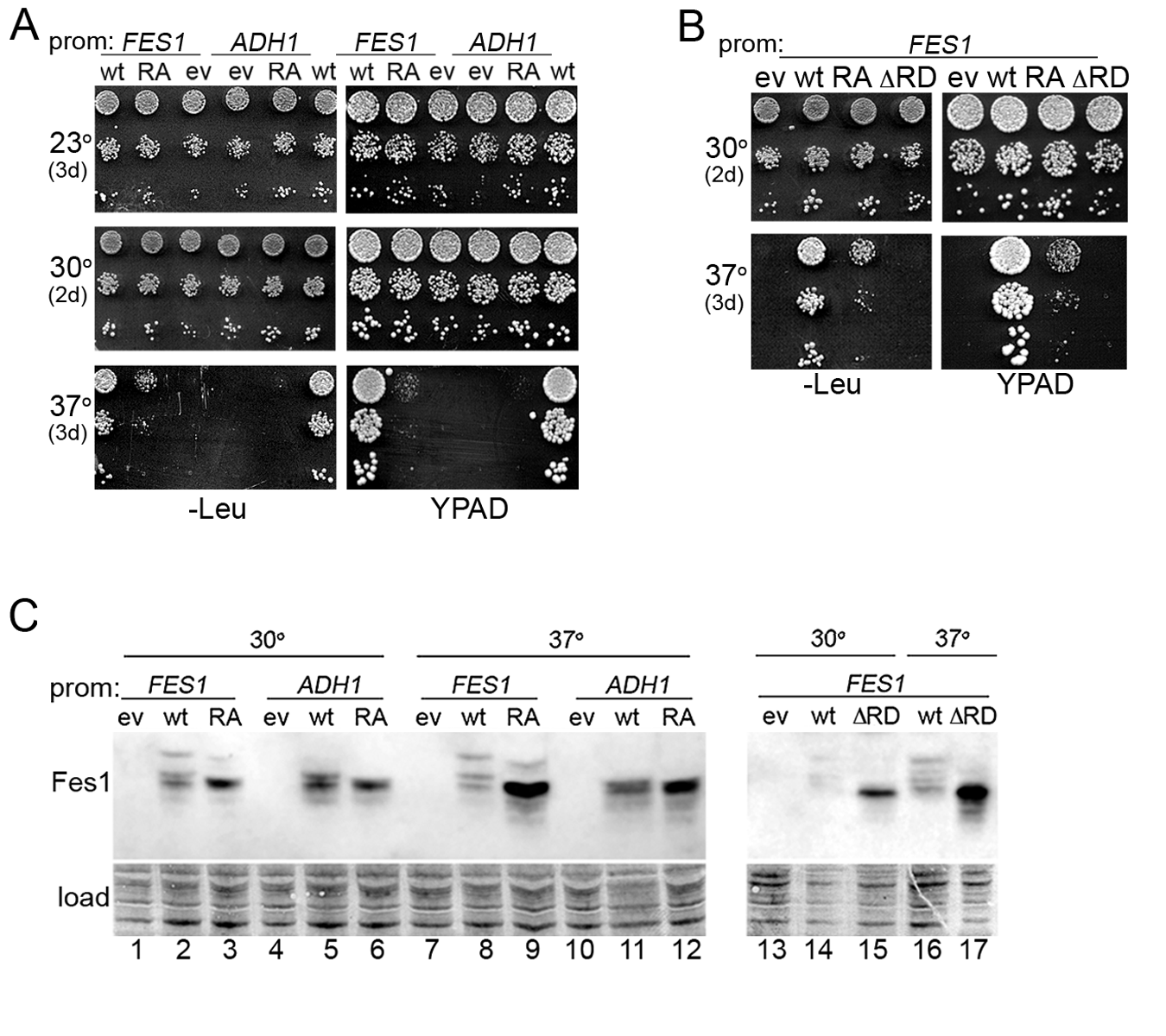

Supplement: S2 Fig — (A) Plasmid transformants of strain 1822 (YPH499 fes1Δ) with empty vector (ev) or the vector encoding Fes1 (wt) or Fes1A79R,R195A (RA) were grown in -Leu liquid medium, diluted, plated on -Leu or YPAD and incubated at indicated temperature for 2–3 days as indicated. Cells express Fes1 from the weak ADH1 promoter [14,30,31] or the native FES1 promoter as indicated (prom). When Fes1A79R,R195A is regulated by the FES1 promoter growth at 37°C is more noticeable. (B) As in (A) except using our fes1Δ strain SY346 and plasmids expressing Fes1ΔRD were included. (C) Western analysis of Fes1 proteins from the same strains as in panel (A) (lanes 1–12) and panel (B) (lanes 13–17), as indicated, grown in liquid YPAD at the indicated temperature. Image labeled "load" shows the blotted membranes stained by amido-black as loading and transfer controls. Growth differences do not seem to be due simply to differences in expression. (TIF) [file pgen.1008219.s002.tif]

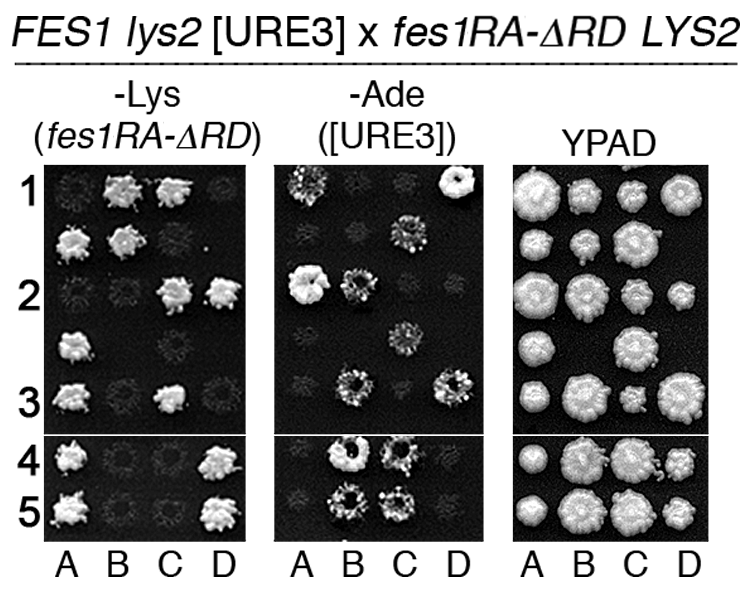

Supplement: S3 Fig — Dissected tetrads of sporulated [URE3] diploids of parents indicated above were replica-plated onto -Lys medium selecting for cells expressing Fes1A79R,R195AΔRD (FES1 is linked to LYS2 on chromosome 2) and medium lacking adenine (-Ade), which selects for cells propagating [URE3] (see text). Five of the tetrads shown (1–5) have four viable spores. On primary dissection plate (YPAD) all Fes1A79R,R195AΔRD colonies are smaller than wild type colonies, which implies the slower growth caused by expression of Fes1 lacking its RD is not due to a growth-inhibitory interaction of Fes1ΔRD with Hsp70. (TIF) [file pgen.1008219.s003.tif]
